# Predictive regression models for cognitive impairment, dementia, and Alzheimer’s disease using real-world electronic health records

**DOI:** 10.3389/fneur.2025.1522340

**Published:** 2025-10-20

**Authors:** Raquel Yubero, Rocío García-Cobos, Elena García-Arcelay, Alicia Algaba, Pablo Rebollo, Jorge Maurino, Rafael Arroyo

**Affiliations:** ^1^Department of Neurology, Hospital Universitario Quirónsalud, Madrid, Spain; ^2^Medical Department, Roche Farma, Madrid, Spain; ^3^IQVIA, Madrid, Spain

**Keywords:** Alzheimer’s disease, dementia, risk factors, cognitive impairment, regression model

## Abstract

The aim of this non-interventional, case–control pilot study was to identify factors associated with cognitive impairment, dementia, and Alzheimer’s disease (AD) using a real-world dataset from Quirónsaludmadrid’s database. Based on Global Deterioration Scale score, 4 models of regression aimed to predict cognitive impairment and dementia (model 1), mild cognitive impairment (MCI, model 2), AD (model 3) and progression (model 4) were created. Age [odds ratio (OR) = 1.721], apathy (OR = 34.952), anxiety (OR = 0.223) and higher education (OR = 0.026) were associated with model 1 with an area under the curve (AUC) of 0.796 and a sensitivity of 0.60 and specificity of 0.86. For model 2, the selected variables were: age (OR = 1.222), apathy (OR = 2.650), depression (OR = 0.318) and higher education (OR = 0.232) with an AUC of 0.657 and a sensitivity of 0.82 and specificity of 0.45. For model 3, variables included were age (OR = 1.490), first-degree family history (OR = 4.147), apathy (OR = 8.247), anxiety (OR = 0.302), and higher education (OR = 0.119) with an AUC of 0.852 and a sensitivity of 0.84 and specificity of 0.73. Model 4 had an AUC of 0.532 and a sensitivity of 0.59 and specificity of 0.65. In conclusion, age and apathy were risk factors for the development of cognitive impairment, MCI and AD, while high education level was a protective factor in the three main models. Family history of dementia was a risk factor for developing AD. Models 3 and 1 had the best selection capacity and could be recommended to predict the diagnosis of AD and cognitive impairment and dementia in individuals with suspicious symptoms or presymptomatic.

## Introduction

The global elderly population is growing significantly. Over the next 15 years, the number of people aged 60 and above will increase by 56% (1). This rapid demographic shift toward an older population will lead to higher rates of disease and disability, notably affecting cognitive functions. Conditions such as mild cognitive impairment (MCI), Alzheimer’s disease (AD), and other types of dementia are expected to become more prevalent as a result (2–6).

Cognitive impairment is defined as a clinical entity characterized by a complete or partial intellectual dysfunction. Given that cognitive impairment is related to age, and that today’s life expectancy is increasing, as has been commented previously, the management of these entities has become a major public health concern that entails a challenge for health and social services and is the main cause of disability and dependence among elderly worldwide (7).

In fact, the total number of people with dementia is expected to reach 82 million in 2030 and 152 million in 2050 according to the World Health Organization (7). Moreover, AD and cognitive impairment have a high burden of disease with a clear impact on morbidity, disability, and mortality (8, 9).

However, despite the high number of cases and high burden of disease, there is a significant percentage of cases that are still underdiagnosed, preventing the early establishment of pharmacological and non-pharmacological treatments that slow cognitive decline and control behavioral disorders (10). Detecting and predicting cognitive decline at its earliest stages is crucial for implementing timely interventions that may help slow disease progression and enhance patient outcomes (11–13).

As cognitive impairment, and specially AD, can be attributed to potentially modifiable risk factors (such as diabetes mellitus, arterial hypertension, obesity, smoking, physical inactivity, depression, cognitive inactivity, and social isolation, among others), the early identification and prevention of these risk factors as well as disease forecasting must be key points to avoid the emergence of new cases. In fact, disease forecasting has been an area of intense interest for the scientific community for over seven decades (14–16) and some groups have developed tools to identify the disease based on patient’s characteristics and medical history (17).

The creation of precise regression models utilizing real-world data presents a promising path toward enhancing our capacity to pinpoint individuals at risk of cognitive impairment and dementia. These models leverage complex datasets encompassing various biological, clinical, and lifestyle factors, enabling the identification of subtle patterns and risk factors that may otherwise go unnoticed (18).

The development of accurate regression models relies on sophisticated machine learning techniques that can analyze large-scale datasets efficiently. These models not only predict future cognitive decline but also provide insights into the underlying mechanisms of disease progression, paving the way for novel therapeutic approaches and precision medicine initiatives (19–21).

So, the primary objective of this pilot study was to develop a regression model for cognitive impairment and dementia to be applied in healthy subjects, using real world data from a cognitive impairment database owned by the Quirónsalud Dementia Team.

Also, 3 additional regression models using the same methodology were developed for the prediction of MCI, AD, and worsening cognitive impairment (exploratory model in patients with several neuropsychological determinations performed over time).

## Materials and methods

### Design

A pilot case–control study for the development of different cognitive impairment regression models to be applied in the future in healthy subjects as a risk calculator was designed. The results for the study were obtained analyzing the database owned by the Quirónsalud Dementia Team. This database contains data from individuals who were assisted by the Neurology Department at Hospital Quirónsalud Madrid between 2007 and 2022 due to cognitive complaints, including determinations such as Global Deterioration Scale (GDS) or Neuropsychiatric Inventory (NPI-Q) scale. Each patient could have more than one assessment, so each assessment was considered as a singular case in the majority of analyses.

The Quironsalud Madrid University Hospital is a private healthcare center in Spain specializing in neurological care and research. The neurology department includes 20 neurologists and two neuropsychologists. Annually, the department handles approximately 50,000 neurological consultations, with about 15% (7,500 consultations) related to cognitive disorders.

The NPI consists of 12 items assessing the presence and severity of 12 neuropsychiatric symptoms (22). All patients with more than 1 point in this questionnaire were considered as a case for this variable in the present study. Regarding GDS score, this score is made up of the following categories: 1. Absence of cognitive impairment; 2. Memory complaints; 3. MCI; 4. Moderate cognitive impairment; 5. Moderate–severe cognitive defect; 6. Severe cognitive impairment; 7. Very severe cognitive defect (23). Based on this score, patients and assessments were classified as controls and cases for the regression models created for this study in the next step: those with a low GDS score, GDS = 1 or 2, were “controls,” while those with higher scores, GDS ≥ 3, were considered “cases.” Within this last category, MCI cases were those with GDS = 3 and AD cases were those with GDS ≥ 3 and neurological clinical diagnosis compatible with AD. This neurological diagnosis was also considered in the database and was based on the International Classification of Diseases.

Individuals with multiple neuropsychological tests determinations during data collection were analyzed in an additional model that aimed to identify worsening cognitive impairment/dementia. In this case there were two groups; patients who worsened their GDS score over time (increase in GDS score of at least one point) and patients who kept or improved it (no increase in GDS score or decrease of at least 1 point). The difference between scores was calculated taking last determination as reference.

These neuropsychological tests, as well as age, educational level, profession, and familiar history of cognitive impairment were obtained during the routine visits of patients to neurologists during the data collection period (between 2007 and 2022), following the standard clinical practice.

Clinical variables and medical history including diabetes mellitus (DM), hypertension, smoking, and alcohol consumption were extracted from electronic medical records for those patients.

The four models proposed to achieve the study’s objectives were as follows:

- One model for cognitive impairment/dementia (model 1): comparison of cases with any cognitive impairment and dementia (GDS ≥ 3) vs. controls (GDS = 1 or 2).
- A second model for MCI (model 2): comparison of cases with MCI (GDS = 3) vs. controls (GDS = 1 or 2).
- A third model for AD (model 3): comparison of cases with AD diagnosis (GDS score ≥ 3 and neurological clinical diagnosis of AD) vs. controls (GDS = 1 or 2).
- A fourth model for cognitive impairment and dementia in patients with multiple neuropsychological test determinations (model 4): comparison of cases who worsen their GDS score over time vs. those who keep or improve it.

All models were constructed from the same database of patient assessments. The analysis of the four models were independenly performed and no comparisons between models were made.

### Population

Assessments of individuals with cognitive complaints between 2007 and 2022 were included in the database. Individuals who were unable to perform the required neurological tests for any reason were excluded. No other criteria were considered as inclusion or exclusion criteria.

This pilot study included a final sample of 2,497 individuals. Of these, there were 24 individuals without a GDS score. The data from these patients were included in the descriptive analysis of demographic characteristics but not in the regression models. Regarding number of assessments, there were 2,996 assessments, 2,965 of them with a GDS score, the remaining 31 that did not present a cognitive assessment were not included in the models.

### Statistical methods

First, a descriptive analysis to understand the characteristics of the sample studied was performed. Continuous variables were reported as mean (and standard deviation) or median and interquartile range where appropriate. Categorical variables were summarized as relative and absolute frequencies. Such descriptive statistics were reported for the total study population, and for each subsample used in each model. No imputation of missing values was performed for any variable. The number of missing values was quantified and provided.

Next, four logistic regression models were developed to identify the predictor variables of the corresponding outcomes. All the models were performed at the level of number of assessments and not at the level of number of individuals. Since the same participant could have different determinations and different score in each, each determination was considered as a singular case.

Logistic regression models were built as Generalized Linear Mixed Models (GLMM). GLMM contain terms to account for both fixed and random effects. When introducing random effects, variance within subjects was considered, therefore several entries from the same subject could enter the model. The use of GLMM allowed the utilization of the entire data set, since it contained patients with multiple entries, providing more complete and precise models. Models were built following the steps below:

- Step 1: Corresponding subset of assessments was extracted from raw data for each model to estimate binary response of presence or absence of dementia (model 1); MCI (model 2) or AD (model 3). For model 4 (exploratory), only patients with more than one measurement were selected: to estimate worsening cognitive impairment based on GDS score (patients with more than one point increase in GDS score).
- Step 2: In all models, a categorical variable was created to discern corresponding controls and cases. For the last model (4), a variable indicating worsening (case) or not (control) was created, based on the difference between the last and the first GDS score.
- Step 3: Data was randomly divided into a training dataset and a test dataset at an approximate ratio of 3:1. The model was developed in the training data set and was later validated in the test dataset.
- Step 4: A multiple logistic regression model was built by selecting the best features for the model through stepwise regression. This is a procedure which enters and removes predictors in a stepwise manner into the model until there is no statistically valid reason to enter or remove any more.
- Step 5: Collinearity of selected variables was tested by using the variance inflation factor.
- Step 6: A multiple logistic regression model was fitted with previously selected variables. If only one covariate was to be included, a simple logistic regression model was used.
- Step 7: Receiver Operating Characteristics (ROC) curve and Area Under the ROC Curve (AUC) were calculated on a training dataset.
- Step 8: To validate the model, it was applied to the test dataset to see if the model predicted well when faced with different data. Discrimination was evaluated by means of ROC and AUC.

An AUC ≥ 0.9 was considered excellent, between 0.8 and 0.9 is good, between 0.7 and 0.8 fair, between 0.6 and 0.7 poor, and between 0.6 and 0.5 was considered a fail (24).

Sensitivity and specificity were reported corresponding to probability thresholds selected by the highest Youden Index.

For all tests, a *p*-value lower than 0.05 was considered significant and *p*-values between 0.05 and 0.1 were considered as trend towards to significance.

## Results

### Studied population

A final sample of 2,497 individuals was included. The total number of assessments was 2,996; of these, 2,965 had a GDS score and 31 did not present cognitive assessment and were not included in the models but were included in the descriptive analysis. Based on GDS score, 623 assessments were cataloged as “cognitive healthy” (controls evaluations), 2,342 as cognitive impairment and dementia (patients included in model 1), 644 as MCI (patients included in model 2), and 966 as AD (these assessments were based on GDS score and clinical diagnosis, patients included in model 3). So, of the 2,342 assessments included in model 1, 644 correspond to MCI and 966 correspond to AD; these were also included in model 2 and 3, respectively. In addition, there were 379 patients that had more than one neuropsychological evaluation (758 assessments, corresponding to the first and the last assessments of these patients).

### Sociodemographic characteristics

The sociodemographic characteristics of the different evaluation groups included in the study are shown in Table 1. The mean age of the whole sample analyzed was 73 years; almost half of evaluated patients (43.6%) had more than 20 years of education and approximately two-thirds (63.3%) were professionals and 17.1% had first-degree family history of dementia (Table 1).

**Table 1:** Sociodemographic data for the four different evaluation groups used to build the regression models.

Variable	Control (*N* = 623)	Dementia (*N* = 2,342)	MCI (*N* = 644)	AD (*N* = 966)	Total evaluations (*N* = 2,996)
Age [Mean (SD), years]	64.4 (10.7)	75.4 (7.8)	72.1 (7.9)	76.8 (7.3)	73.0 (9.6)
Education [*n* (%)]					
<5 years	4 (0.6%)	47 (2.0%)	8 (1.2%)	26 (2.7%)	52 (1.7%)
5–10 years	2 (0.3%)	73 (3.1%)	14 (2.2%)	33 (3.4%)	75 (2.5%)
11–15 years	103 (16.5%)	715 (30.5%)	149 (23.1%)	330 (34.2%)	822 (27.4%)
16–20 years	154 (24.7%)	574 (24.5%)	167 (25.9%)	229 (23.7%)	740 (24.7%)
>20 years	360 (57.8%)	932 (39.8%)	306 (47.5%)	348 (36.0%)	1,306 (43.6%)
Profession [*n* (%)]					
Manual	10 (1.6%)	109 (4.7%)	21 (3.3%)	60 (6.2%)	119 (4.0%)
Technical	17 (2.7%)	71 (3.0%)	23 (3.6%)	26 (2.7%)	88 (2.9%)
Professional	475 (76.2%)	1,399 (59.7%)	423 (65.7%)	502 (52.0%)	1,897 (63.3%)
Manager	34 (5.5%)	105 (4.5%)	40 (6.2%)	49 (5.1%)	139 (4.6%)
Military	15 (2.4%)	51 (2.2%)	15 (2.3%)	12 (1.2%)	67 (2.2%)
N/A	72 (11.6%)	607 (25.9%)	122 (18.9%)	317 (32.8%)	686 (22.9%)
Smoking status [*n* (%)]					
Yes	47 (7.5%)	116 (5.0%)	44 (6.8%)	43 (4.5%)	164 (5.5%)
Alcohol consumption [*n* (%)]					
Yes	8 (1.3%)	63 (2.7%)	16 (2.5%)	23 (2.4%)	72 (2.4%)
First degree history of dementia [*n* (%)]					
Yes	132 (21.2%)	374 (16.0%)	114 (17.7%)	193 (20.0%)	513 (17.1%)

AD, Alzheimer’s disease; MCI, mild cognitive impairment; SD, standard deviation; N/A, not available.

By groups, the control group had the lowest mean age (64.4 years) and the highest level of education (99.0% of entries were from subjects who had studied for 11 years or more). By contrast,the AD group had the highest mean age (76.8 years) and fewer years of education (93.9% of group entries represented patients that had studied for 11 years or more, being the smallest percentage compared to the same categories in other groups). Variables corresponding to profession, smoking status and alcohol consumption presented similar distribution across all groups. Regarding family history, controls (21.2%) and AD (20.0%) patients were those with the highest percentage of first-degree history of dementia (Table 1).

### Regression models

The results obtained for the different regression models were as follows:

- For model 1 (cognitive impairment and dementia) the selected predictive variables were: age (OR = 1.721), apathy (OR = 34.952), anxiety (OR = 0.223) and education [OR = 0.024 (16–20 years) and 0.026 (>20 years) vs. ≤15 years] with an AUC of the ROC curve of 0.796 and a sensitivity of 0.60 and specificity of 0.86.
- For model 2 (MCI), the selected variables were: age (OR = 1.222), apathy (OR = 2.650), depression (OR = 0.318) and education [OR = 0.232 (16–20 years) and 0.217 (>20 years) vs. ≤15 years] with an AUC of the ROC curve of 0.657 and a sensitivity of 0.82 and specificity of 0.45.
- For model 3 (AD), the variables included were age (OR = 1.490), family history (OR = 4.147 first degree vs. none), apathy (OR = 8.247), anxiety (OR = 0.302), and education [OR = 0.103 (16–20 years) and 0.119 (>20 years vs. ≤15 years)] with an AUC of the ROC curve of 0.852 and a sensitivity of 0.84 and specificity of 0.73.
- For model 4 (worsening cognitive impairment and dementia) only age was selected (OR = 1.003) with an AUC of the ROC curve of 0.532 and a sensitivity of 0.59 and specificity of 0.65.

The estimated parameters of probability’s distribution for each of the 4 models are described in Table 2.

**Table 2:** Parameters of probability’s distribution for the regression models.

Model	Analysis of maximum likelihood estimation						
	Parameter	Coefficient	Standard error	*p*-value	OR	95%CI OR	VIF
Model 1 cognitive impairment/dementia	Intercept	−29.5419	3.0089	<0.0001	–	–	–
	Anxiety	−1.5014	0.6524	0.0223	0.223	0.062–0.806	1.04821
	Apathy	3.5540	0.6536	<0.0001	34.952	9.642–126.704	1.04920
	Age	0.5431	0.04385	<0.0001	1.721	1.579–1.877	1.03833
	Education (16–20 vs. 0–15 years)	−3.7477	0.9402	<0.0001	0.024	0.004–0.150	1.03275
	Education (> 20 vs. 0–15 years)	−3.6324	0.8177	<0.0001	0.026	0.005–0.132	
Model 2 mild cognitive impairment	Intercept	−12.1441	1.9266	<0.0001	–	–	–
	Depression	−1.1447	0.4315	0.0106	0.318	1.134–0.757	1.37536
	Apathy	0.9747	0.4364	0.0299	2.650	1.104–6.364	1.35099
	Age	0.2004	0.02664	<0.0001	1.222	1.158–1.289	1.04757
	Education (16–20 vs. 0–15 years)	−1.4606	0.6012	0.0187	0.232	0.069–0.776	1.07999
	Education (> 20 vs. 0–15 years)	−1.5277	0.5472	0.0074	0.217	0.072–0.651	
Model 3 Alzheimer disease	Intercept	−26.1965	2.4343	<0.0001	–	–	–
	Apathy	2.1099	0.4428	<0.0001	8.247	3.416–19.913	1.05904
	Age	0.3989	0.03362	<0.0001	1.490	1.394–1.593	1.06661
	Anxiety	−1.1974	0.4361	0.0075	0.302	0.127–0.720	1.6876
	Family history (1st degree vs. None)	1.4224	0.5375	0.0098	4.147	1.423–12.091	1.00643
	Family history (1st degree vs. none)	−0.05578	0.9572	0.9537	0.946	0.141–6.359	
	Family history (1st degree vs. none)	4.1139	2.3047	0.0782	61.184	>999.999	
	Education (16–20 vs. 0–15 years)	−2.2751	0.5909	0.0002	0.103	0.032–0.333	1.06236
	Education (>20 vs. 0–15 years)	−2.1270	0.5179	<0.0001	0.119	0.043–0.344	
Model 4 worsening in cognition	Intercept	−15.4738	1.1641	<0.0001	–	–	–
	Age	0.03248	0.01561	0.0386	1.033	1.002–1.065	–

CI, Confidence interval; OR, Odd ratio; VIF, Variance Inflation Factor.

**Figure 1:**
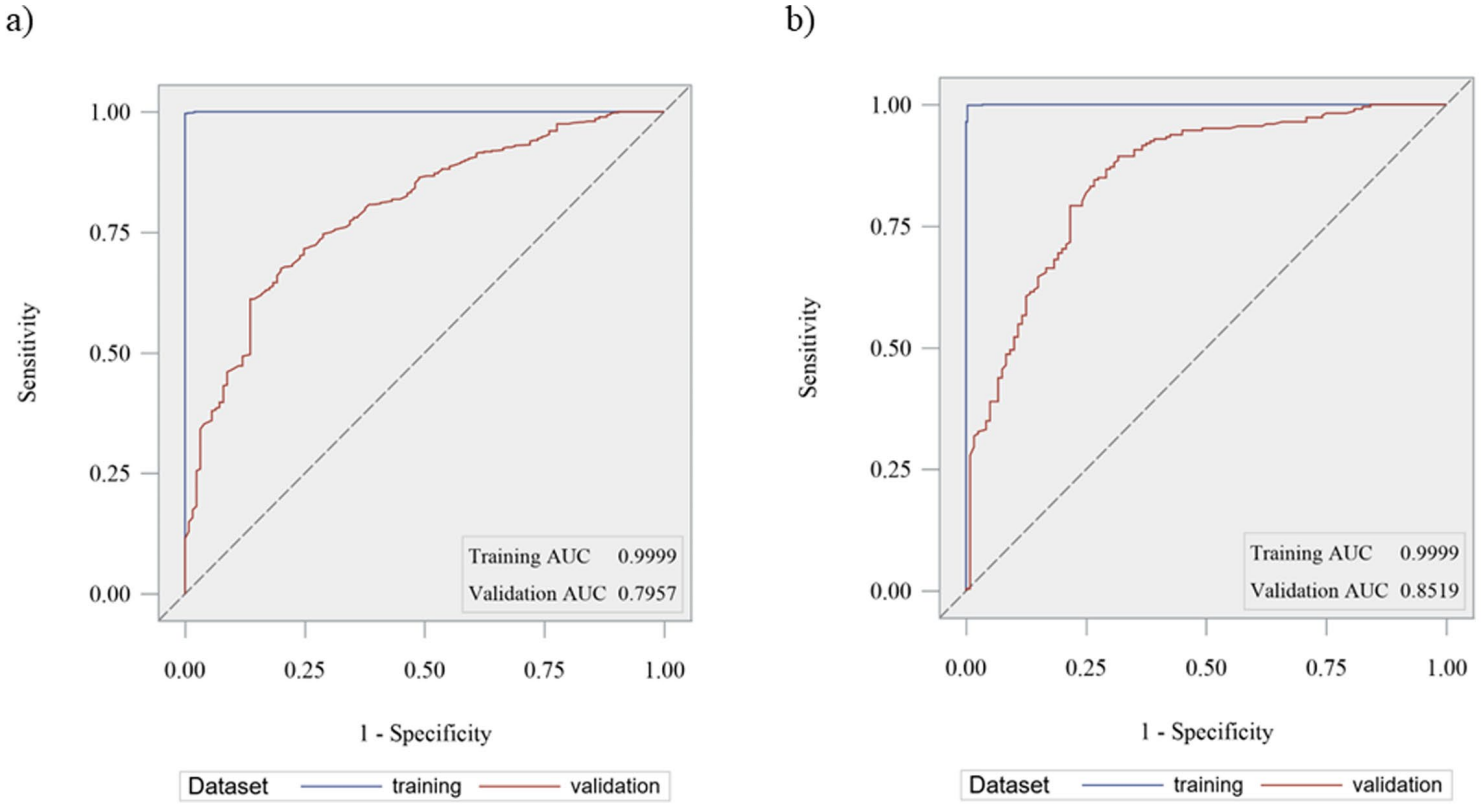
Area under the curve (AUC) for Receiver Operating Characteristics (ROC) curves for model 1 **(a)** and model 3 **(b)**.

Model 3 showed the best selection capacity (AUC 0.85) followed by model 1 (AUC 0.80). On the contrary, model 4 demonstrated the poorest selection ability (AUC 0.53), followed by model 2 (AUC 0.67). Figure 1. shows the AUC for ROC curves for model 1 (Figure 1a) and model 3 (Figure 1b).

## Discussion

Cognitive impairment, particularly AD, can often be linked to potentially modifiable risk factors, such as diabetes mellitus, arterial hypertension, obesity, smoking, physical inactivity, depression, cognitive inactivity, and social isolation (11). Other recent publications also have highlihted other factors such as untreated vision loss, osteoporosis or high LDL cholesterol, as risk factors for dementia (25, 26). Early identification and prevention of these risk factors, along with accurate disease forecasting, are essential strategies to prevent new cases. Disease forecasting has been a significant focus of the scientific community for over 70 years, and various groups have developed tools to identify the disease based on patients’ characteristics and medical history.

Age, low educational level, and apathy were the most important risk factors in the main models analyzed in our study. It is known that aging is the most powerful risk factor for the development of many chronic diseases including dementia, due to the alteration of numerous cellular and molecular pathways (27). It has been described that adaptation to stress, epigenetic, inflammation, macromolecular damage, metabolism, proteostasis, stem cells, regeneration and defective autophagy may be considered the main cellular and molecular mechanisms that underpin the aging process (28). Individuals with a higher level of education had a lower risk of development of cognitive impairment and dementia (OR = 0.024), MCI (OR = 0.232) or AD (OR = 0.103), in line with previous studies. Educational attainment has long been linked for to an increased cognitive function over the lifespan and to a lowered risk of dementia (29–31). Education level is related to cognitive abilities such as psychomotor speed, memory, and abstract reasoning. Some authors have found that the development improvement of these cognitive abilities during the first decades of life carries great potential for improving cognitive ability in early adulthood and persist into older age (32). Moreover, cognitive training intervention can decrease the deterioration of cognitive function once the diagnosis of MCI has been performed and can help to delay the progression to other dementias (33). This is because cognitive training could stimulate pre-existing neural reserves or recruit neural circuitry as “compensatory scaffolding” prompting neuroplastic reorganization as an adaptive response (34, 35). In our sample, almost half of patients had more than 20 years of education, denoting a highly educated patient population.

Apathy and anxiety were also predictive variables in our study. Nevertheless, while apathy was a risk factor for all three models (OR model 1 = 34.952; OR model s.650; OR model 3 = 8.247), anxiety was revealed as a protective factor for models 1 (cognitive impairment and dementia; OR = 0.223) and 3 (AD; OR = 0.302). In addition, depression was a protective factor in regression model 2 (OR = 0.318).

Depression, anxiety, and apathy are neuropsychiatric features commonly observed in MCI (36–39). Some publications have described that in subjects with MCI, symptoms of anxiety, agitation and irritability may reflect underlying AD pathology. Ramakers et al. found that patients with symptoms of anxiety had abnormal cerebrospinal fluid amyloid-β 42 (OR = 2.3) and t-tau (OR = 2.6) concentrations with respect to patients with normal cognitive status (40). Although anxiety may be a psychological reaction to the insight into their cognitive decline, or induce a hypothalamic–pituitary–adrenal axis dysregulation in AD pathology (35, 41), other studies in line with our findings did not find this association, considering the anxiety as a non-predictor for conversion to AD (42). The justification for these results is not easy, but probably one explanation could be that once the cognitive impairment progresses, patients could lose their objective perception of memory deficits and symptoms and their anxiety levels would go down. Also, because the use of anxiolytic and antidepressant treatments is common in this population group, its use could influence the NPI scores obtained, so that the symptoms could be under control with the treatment received at the time of neurological assessment.

Regarding the influence of depression in MCI and dementia, the results published are also discrepant, as other authors in line with our results did not find an association between depressive symptoms and AD (40, 43, 44). In contrast, other studies have reported that depressive symptoms predicted cognitive decline and AD in subjects with MCI (45, 46). Because depressive symptoms in subjects with MCI may be related to other neurodegenerative processes, such as synaptic or neuronal loss, vascular changes, neurotransmitter deregulation or primary affective disorder (47, 48), further studies would be necessary to elucidate its role in the MCI and dementia. As mentioned above, it would also be important to know the influence that antidepressant treatments may have had on the NPI scores obtained, since the study population is a population with a high demand for treatment.

On the other hand, apathy may be the result of the degeneration of frontal circuits and white-matter lesions, and more severe cholinergic dysfunction (49, 50). Recent studies associated apathy with incident dementia and worse clinical outcomes (cognition, function, neuropsychiatric symptoms, and caregiver burden) considering this symptom a marker of clinical decline in older people and poorer outcomes across neurocognitive disorders (51). In addition, apathy has been associated with an increased risk of conversion to AD in patients with MCI (52). Considering all these findings, the evaluation of this variable must be key to predicting the diagnosis of MCI and conversion to AD and other dementias and its therapeutic approach must be considered once the diagnosis is confirmed.

In model 3 (AD), in addition to age, education, apathy and anxiety, family history was also considered a risk factor for developing the disease. It has been previously published that the heritability in this pathology is high, it has been estimated that up to 60–80% of patients with AD have previous family history (53). Although numerous studies are still being carried out, this strong genetic component is widely accepted, and recent studies have detected up to 73 independent loci that could be implied in developing the of disease (54). Therefore, this factor must be taken into account when a dementia diagnosis is performed, and must be a key factor to be included in a model for the early detection of presymptomatic AD.

Cognitive impairment, and specially AD, can be attributed to other potentially modifiable risk factors. In fact, hypertension, high cholesterol, diabetes, and smoking at midlife are each associated with a 20 to 40% increased risk of dementia (55–58). Although the control of these factors is recommended, and lifestyle modification is always a strategy for preventing of different complications, in our study no association with vascular risk factors was found. It may have been because these variables were directly extracted from medical records, and they were not collected at the time of neuropsychological tests performing. These results should be taken with caution since they could be underestimated, because existing medical chart data might not contain all the information required or might not be up to date.

As previously mentioned, cognitive decline is usually progressive going from different phases ranging from subjective cognitive impairment (cognitive complaint with normal cognitive screening test) to MCI to dementia (mostly in the form of AD) (59). In the present study, an exploratory model was created in order to predict worsening cognitive impairment/dementia (model 4). In this model, a comparison between patients who worsen their GDS score over time vs. those who keep or improve it was made, however a poor sensitivity (0.59) and specificity (0.65) and, therefore, a poor selection capability, was obtained. Thus, additional studies with systematic and protocoled evaluations in this population should be performed to obtain data that are more conclusive.

According to the results of AUCs obtained in our study, model 3 (AD) was the model with the best selection capacity with an AUC of 0.85 followed by model 1 (cognitive impairment and dementia that obtained a good selection performance model with an AUC of 0.80). Its use, therefore, could be recommended to predict the diagnosis of cognitive impairment and dementia (including AD) in healthy individuals who go to the clinic after or before the appearance of suspected symptoms. Based in our results, the age, education level, apathy and anxiety could be key factors to include in both models. In addition, family history could be also considered in the AD model.

By contrast model 4 (worsening cognitive impairment and dementia) demonstrated the poorest selection ability with an AUC of 0.53, followed by model 2 (MCI) that was also considered poor with an AUC of 0.66 its clinical application could not be recommended for the time being.

This study has several limitations. Key variables such as smoking, alcohol consumption, diabetes mellitus, and hypertension were directly extracted from electronic medical records rather than being collected contemporaneously with the neuropsychological assessments. These factors might be underestimated due to incomplete or inconsistent documentation in medical charts, which can vary according to the practice patterns of different specialists. In our sample, almost half of the patients had more than 20 years of education, denoting a highly educated patient population, in concordance with the type of patient followed in a private healthcare setting, with more socioeconomic resources and possibility of academic formation. Also, probably the age at which the patient consults as first time may be different in a private compared to public setting, patients would go to private healthcare earlier to evaluate neurological symptoms since access to the specialist could be faster. So, the results obtained to this regard may not be directly generalizable to other populations. Therefore, caution should be exercised when interpreting the results, and the possibility of conducting additional studies with more diverse samples should be considered.

Because the database used is the same for all the models, patients included in the more general model 1 could overlap with those patients included in the more specific models 2 and 3. Since the comparison between models was not the objective of study and the clinical significance and utility of these models as well as interpretation were different, no interferences due to this fact were estimated. Also, although all-cause dementia cases were included in model 1 and it was our population of interest in the study, a separate model excluding MCI and AD could have helped to elucidate if the key predictors detected in all-cause model 1 remained significant and consistent once the subtypes were removed. However, because majority of cases included in model 1 corresponded to MCI and AD and this model was not the scope of study, the analysis of this additional model was not finally performed.

Additionally, in patients with longitudinal data, follow-up visits were scheduled based on individual patient needs rather than a standardized protocol. This variability could affect the consistency and reliability of the data. Moreover, both the physicians and patients involved in the study may not be fully representative of all specialists and individuals with cognitive impairment or dementia in Spain, as the sample was drawn from a private healthcare setting. Finally, despite these limitations, this study provides valuable insights into these conditions in a real-life context, given the lack of previous similar data in our region. Also, the models use variables that are easy to extract from computerized medical records, making possible to apply them in any healthcare setting for the early detection of cases at risk of cognitive impairment that could be subject to more intensive monitoring.

## Conclusion

Our study highlights the significance of age, education level, and apathy as key risk factors for cognitive impairment and AD. While anxiety and depression presented mixed associations, our findings emphasize the protective role of higher educational attainment against cognitive decline. Notably, apathy emerged as a consistent risk factor across various models, underscoring its importance in predicting the progression of cognitive impairment. Family history also contributed to the risk of AD, aligning with the recognized genetic predisposition in this pathology. The robust performance of our AD prediction model (AUC of 0.85) and the cognitive impairment and dementia model (AUC of 0.80) supports their potential utility in clinical settings. Conversely, models predicting the progression of cognitive impairment and MCI demonstrated limited predictive capability, indicating the need for further research.

The integration of age, education level, apathy, and anxiety into predictive models offers a promising approach for early identification and intervention in cognitive impairment and AD. Future studies should focus on systematic and standardized data collection to enhance the reliability and applicability of these predictive tools.

## Data Availability

The raw data supporting the conclusions of this article will be made available by the authors, without undue reservation.
